# Supplementary material for: Assessment of knowledge, practice, and status of food handlers toward Salmonella, Shigella, and intestinal parasites: A cross-sectional study in Tigrai prison centers, Ethiopia
Source: PLoS One. 2020 Nov 3;15(11):e0241145. doi: 10.1371/journal.pone.0241145 (PMC7608870; doi:10.1371/journal.pone.0241145)
Supplement: S1 File — (ZIP) [file pone.0241145.s001.zip › Consent Form for prisoners.docx]

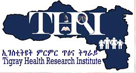


**The Government of National regional State of Tigray, Tigray Health Research Institute**

This Informed consent form is for the study participants in Tigrai prison centers who we are inviting to participate in the research entitled **“**Assessment of knowledge, practice, and status of food handlers toward Salmonella, Shigella, and intestinal parasites: A cross-sectional study in Tigrai prison centers, Ethiopia, 2019**’’**

Name of principal investigator: Fitsum Mardu Landu

Name of organization: Adigrat University, Ethiopia

Name of sponsor: Adigrat University, Ethiopia

**Name of proposal**: Assessment of knowledge, practice, and status of food handlers toward Salmonella, Shigella, and intestinal parasites: A cross-sectional study in Tigrai prison centers, Ethiopia, 2019

This Informed Consent Form has two parts:

• Information Sheet (to share information about the study with you)

• Certificate of Consent (for signatures if you choose to participate)

You will be given a copy of the full Informed Consent Form

**Part I: Information Sheet**

**Introduction**

I am Fitsum Mardu, working for the Adigrat University. We are doing research on Salmonella, Shigella, and intestinal parasites, which are very common in this country and in this region. We are going to give you information and invite you to be part of this research. You do not have to decide today whether or not you will participate in the research. Before you decide, you can talk to anyone you feel comfortable with about the research. This consent form may contain words that you do not understand. Please ask me to stop as we go through the information and I will take time to explain. If you have questions later, you can ask them of me or of another researcher.

**Purpose of the research**

Salmonella, Shigella, and intestinal parasites are the main causes of sickness and death of many people in Ethiopia. We want to assess the level of knowledge, attitude, and status of prisoners towards these microorganisms which is important for implementing interventions. We believe that you can help us by responding to the questions and giving stool specimen.

**Type of research intervention**

In this research you will participate in an interview regarding your knowledge and practice towards the prevention of Salmonella, Shigella, and intestinal parasites. We also need you to give stool specimens for microbiological examination.

**Participant selection**

We are inviting all prisoners in your institution to participate in this study.

**Voluntary participation**

Your participation in this research is entirely voluntary. It is your choice whether to participate or not. If you choose not to participate all the services you receive at this institution will continue and nothing will change.

**Procedures**

We are inviting you to take part in this research. If you accept, you will be asked to take part in an interview to ask your knowledge and practice towards the aforementioned organisms. You will also be asked to bring a piece of stool specimen once for laboratory examination. The stool specimens will be used only for this research, and will be destroyed when the research is completed. We can also answer questions about the research that you might have.

**Duration**

The research takes place over six months in total. During that time, we will visit you once, for 30 minutes, for both the interview and stool examination.

**Risks**

There is no risk that will happen to you by participating in this research.

**Benefits**

There will be no direct benefit to you, but your participation is likely to help the government of Ethiopia or Tigray regional state to prevent and control Salmonella, Shigella, and intestinal parasites.

**Reimbursements**

You will not be provided any incentive or gift to take part in the research.

**Confidentiality**

The information that we collect from this research will be kept confidential. Information about you that will be collected during the research will be put away and no-one but the researchers will be able to see it. Any information about you will have a number on it instead of your name. Only the researchers will know what your number is and we will lock that information up with a lock and key.

**Sharing the results**

Nothing that you tell us today will be shared with anybody outside the research team, and nothing will be attributed to you by name. The knowledge that we get from this research will be shared with you and your community before it is made widely available to the public. Each participant will receive a summary of the results. We will publish the results so that other interested people may learn from the research.

**Right to refuse or withdraw**

You do not have to take part in this research if you do not wish to do so, and choosing to participate will not affect your treatment at this institution in any way. You will still have all the benefits that you would otherwise have at this prison. You may stop participating in the research at any time that you wish without losing any of your rights as a prisoner here.

**Who to contact**

If you have any questions, you can ask now or later, even after the study has started. If you wish to ask questions later, you may contact:

**Fitsum Mardu Landu**

Phone: 0914366474

Email: [fmardu25@gmail.com](mailto:fmardu25@gmail.com)

This proposal has been reviewed and approved by Institutional Review Board of Tigray Health Research Institute, which is a committee whose task it is to make sure that research participants are protected from harm. If you wish to find about more about the IRB, contact the office:

Phone: +251(0)342411955

Hawelti Subcity, Adishumduhun, P.O.Box: 1547

Mekelle, Tigray, Ethiopia

**Part II: Certificate of Consent**

**I have read the foregoing information, or it has been read to me. I have had the opportunity to ask questions about it and any questions I have been asked have been answered to my satisfaction. I consent voluntarily to be a participant in this research**

**Print Name of Participant__________________**

**Signature of Participant ___________________**

**Date ___________________________**

**Day/month/year**

**I have witnessed the accurate reading of the consent form to the potential participant, and the individual has had the opportunity to ask questions. I confirm that the individual has given consent freely.**

**Print name of witness____________ Thumb print of participant**

**Signature of witness _____________**

**Date ________________________**

**Day/month/year**

**Statement by the researcher/person taking consent**

**I have accurately read out the information sheet to the potential participant, and to the best of my ability made sure that the participant understands that the following will be done:**

**1.**

**2.**

**3.**

**I confirm that the participant was given an opportunity to ask questions about the study, and all the questions asked by the participant have been answered correctly and to the best of my ability. I confirm that the individual has not been coerced into giving consent, and the consent has been given freely and voluntarily.**

**A copy of this ICF has been provided to the participant.**

**Print Name of Researcher****/person taking the consent________________________**

**Signature of Researcher /person taking the consent__________________________**

**Date ___________________________**

Day/month/year
