# Supplementary material for: Assessment of knowledge, practice, and status of food handlers toward Salmonella, Shigella, and intestinal parasites: A cross-sectional study in Tigrai prison centers, Ethiopia
Source: PLoS One. 2020 Nov 3;15(11):e0241145. doi: 10.1371/journal.pone.0241145 (PMC7608870; doi:10.1371/journal.pone.0241145)
Supplement: S1 File — (ZIP) [file pone.0241145.s001.zip › Protocol.docx]

**Laboratory Protocol**

Protocol to the manuscript entitled Assessment of knowledge, practice, and status of food handlers toward Salmonella, Shigella, and intestinal parasites: A cross-sectional study in Tigrai prison centers, Ethiopia, 2019

This protocol has been provided by the authors to give readers additional information about the laboratory examination of intestinal parasites and bacterial isolates from stool specimens

1. **Laboratory Diagnosis of Intestinal Parasitic Infections**

Laboratory diagnosis of intestinal parasitic infections can be carried out by detection and identification of the parasites or their particular stages (ova/egg, cyst, larva or trophozoite) in the stool specimen. For this study we used 10% (v/v) formalin solution to maintain the morphological characteristics of parasites.

- 1. **Direct wet mount examination**

Reagents and equipment used

- Normal Saline (0.85% NaCl)
- Lugol’s Iodine
- Glass microscope slides
- Cover slips (22 mm by 22 mm size)
- Pipettes
- Examination Gloves
- Microscope
- Applicator wooden stick

**Procedure**

1. We mixed about 2 mg (the size of match stick head) of stool sample with a drop of saline and a drop of iodine placed on a slide
2. Using an applicator stick, we have removed any gross fibers and fecal particles
3. We then carefully covered the stool with a cover slip to avoid bubble formation
4. Finally, the slides have been initially examined microscopically with the low power objective (10×) and low light. Then, the 40× objective for has been been used to confirm the presence of parasites.

##### **Formalin-Ethyl Acetate Sedimentation Concentration**

Reagents and equipment

- Formol-water, 10% v/v
- Diethyl ether
- Tea strainer
- Centrifuge

**Procedure**

1. About 1 g (pea-size) specimen has been mixed with 4 ml of 10% formol-water well.
2. Then, we added a further of 3 ml 10% formalin solution
3. We then strained the fecal suspension through the tea strainer into a 15 ml conical centrifuge tube.
4. Centrifuged at 500 × g for 10 minutes and decant supernatant.
5. Add 10 ml of 10% formalin to the sediment and mix thoroughly with wooden applicator sticks.
6. Add 4 ml of ethyl acetate, stopper the tube, and shake vigorously in an inverted position for 30 seconds. Carefully remove the stopper.
7. Centrifuge at 500 × g for 10 minutes.
8. Free the plug of debris from the top of the tube by ringing the sides with an applicator stick. Decant the top layers of supernatant.
9. Use a cotton-tipped applicator to remove debris from sides of the centrifuge tube.
10. Add several drops of 10% formalin to resuspend the concentrated specimen. Proceed with applicable testing.
11. **biochemical identification of Shigella and Salmonella**

**Test principles**

Isolation and identification remain the gold-standard for the diagnosis of infections due to *Salmonella* and *Shigella.* Theoretically, culture is 100% specific and unlike rapid tests, yields an isolate which may be subjected to further characterization (e.g. antimicrobial susceptibility testing).

Virtually all isolation protocols for *Salmonella spp.* and *Shigella spp*. include the use of selective and differential media to enhance recovery of the targeted organisms. Selective media are formulated to suppress background flora. These media also provide preliminary, macroscopic, differentiation of enteric organisms on the basis of colony color and morphology.

When interpreting *Salmonella* and *Shigella* cultures, it is important to remember that colony morphology on selective agar is not diagnostic. Colony morphology is used simply as a means to identify colonies for additional testing. Colonies that produce *Salmonella*-like or *Shigella*-like morphology (“suspect colonies”) on selective agar must be subjected to additional biochemical (and serological) testing to confirm the identification.

As other *Enterobacteriaceae* may look similar to *Salmonella* or *Shigella* on selective and media, the presence of suspect colonies alone cannot be considered diagnostic. A final genus / species level identification requires additional testing for confirmation. Similarly, isolates presumptively identified as suspect-*Salmonella spp*. or suspect-*Shigella spp*. on the basis of agglutination with polyvalent antisera must be subjected to biochemical confirmation. Similarly, polyvalent antisera can be a useful screening tool; however, some O and H antigenic types are found in multiple genera among the *Enterobacteriaceae*, so reaction with any given antiserum without adequate biochemical testing is not diagnostic.

- 1. **Salmonella Shigella (SS) Agar**

**Principle**

The presence of bile salts mixture and dyes (brilliant green) inhibits the growth of gram-positive species to a varying degree. Differentiation of enteric organisms is achieved by the incorporation of lactose in the medium. Organisms which ferment lactose produce acid which, in the presence of the neutral red indicator, results in the formation of red/pink colonies. Lactose non-fermenters form colorless colonies. The latter group contains the majority of the intestinal pathogens, including Salmonella and Shigella. The sodium thiosulfate and ferric citrate enable the detection of hydrogen sulfide production as evidenced by colonies with black centers.

**Preparation**

- Suspend 60.0 grams of *Salmonella Shigella* Agar in 1000 ml distilled water.
- Mix well
- Heat to boiling with frequent agitation to dissolve the medium completely.
- Mix well and pour into sterile Petri plates.
- Let the agar solidify and store in the refrigerator (avoid freezing).  Prepared culture media can be kept for at least a week in refrigeration.

**Procedure**

1. Allow the plates to warm to room temperature and the agar surface to dry before inoculating.
2. Heavily inoculate and streak the specimen as soon as possible after collection.
3. If the specimen to be cultured is on a swab, roll the swab over a small area of the agar surface.
4. Streak for isolation with a sterile loop.
5. Incubate plates aerobically at 36 **°**C 24 hours.
6. Examine colonial morphology.

**Interpretation of results**

**Lactose fermenter:**If lactose fermentation occurs, the medium will turn red due to the acidic pH

**Non-Lactose fermenter**: Salmonella and Shigella appear as transparent or translucent colorless colonies. Colonies of *Salmonella spp.* may appear with or without black centers (depending on the species isolated).

**Control species**

Salmonella typhimurium ATCC 14028 = Colorless colonies with black center

Shigella flexneri ATCC 23354 = Colorless colonies

1. **Biochemical tests**
   1. **Triple Sugar Iron Agar (TSI)**

**Composition**

| **Ingredients** | **Composition** |
| --- | --- |
| Beef extract | 3.0 g/l |
| Yeast extract | 3.0 g/l |
| Peptone | 20.0 g/l |
| Glucose | 1.0 g/l |
| Lactose | 10.0 g/l |
| Sucrose | 10.0 g/l |
| Ferrous sulfate or ferrous ammonium sulfate | 0.2 g/l |
| NaCl | 5.0 g/l |
| Sodium thiosulfate | 0.3 g/l |
| Phenol red | 0.024 /l g |
| Agar | 13.0 g/l |
| Distilled water | 1,000 mL |

- **0.1% glucose**: If only glucose is fermented, only enough acid is produced to turn the butt yellow.  The slant will remain red
- **1.0 % lactose/1.0% sucrose:**  If lactose or sucrose or both are fermented, a large amount of acid will produce which turns both butt and slant yellow. So, the appearance of yellow color in both slant and butt indicates that the isolate has the ability to ferment lactose or sucrose or both.
- **Iron**(ferrous sulfate): Indicator of H_2_S formation
- **Phenol red:** Indicator of acidification (It is**yellow in acidic condition** and red under alkaline conditions).
- It also contains **peptone** which acts as a source of nitrogen. (Remember that whenever peptone is utilized under aerobic condition ammonia is produced)

**Preparation of TSI agar medium**

1. Combine the ingredients, and adjust the pH to 7.3
2. Boil to dissolve the agar and dispense into tubes.
3. Sterilize by **autoclaving at 121 °C for 15 minutes**
4. Cool in a slanted position to give a 2.5 cm butt and a 3.8 cm slant.

**Interpretation of Triple Sugar Iron Agar Test**

1. If lactose (or sucrose) is fermented, a large amount of acid is produced, which turns the phenol red indicator yellow both in the butt and in the slant. Some organisms generate gases, which produces bubbles/cracks on the medium.
2. If lactose is not fermented but the small amount of glucose is, the oxygen-deficient butt will be yellow (remember that butt has comparatively more glucose than slant i.e. more media more glucose), but on the slant the acid produced (less acid produces in slant as media in slant is less) will be oxidized to carbon dioxide and water by the organism and the slant will be red (alkaline or neutral pH).
3. If neither lactose/sucrose nor glucose is fermented, both the butt and the slant will be red. The slant can become a deeper red-purple (more alkaline) as a result of the production of ammonia from the oxidative deamination of amino acids (remember peptone is a major constituent of TSI agar).
4. if H_2_S is produced, the black color of ferrous sulfide is seen.

| **Organism** | **Slant** | **Butt** | **Gas** | **H_2_S** |
| --- | --- | --- | --- | --- |
| Shigella | Alkaline (K) | Acid (A) | Neg (-) | Neg (-) |
| Salmonella | Alkaline (K) | Acid (A) | Pos (+) | Pos (+) |

- 1. **Motility-Indol-Ornithine Agar (MIO)**

**Composition**

| **Ingredients** | **Composition** |
| --- | --- |
| Pancreatic Digest of Gelatin | 10.0 gm |
| Pancreatic Digest of Casein | 10.0 gm |
| L-Ornithine | 5.0 gm |
| Yeast Extract | 3.0 gm |
| Dextrose | 1.0 gm |
| Bromocresol Purple | 0.02 gm |
| Agar | 2.0 gm |

**Procedure**

- 1. Allow medium to warm to room temperature prior to inoculation.
  2. Using a straight needle, select isolated colonies from a pure 18-24 hours culture and stab the center of the medium to about one-half its length.
  3. Incubate the tubes aerobically at 35 degrees C. for 18-24 hours.
  4. Caps should be loose during incubation.
  5. Examine for motility and ornithine production

**Interpretation of results**

**Motility**: Positive motility is denoted when turbidity or cloudy growth extends from the line of inoculation. Growth only along the stab line is indicative of a negative motility test.

**Indole test**: A positive test for indole is denoted when a pink to red color band is formed at the top of the medium after addition of Kovacs Reagent. A yellow color denotes a negative indole test after addition of Kovacs Reagent.

**Ornithine test**: A positive test for ornithine is denoted by a dark, turbid purple color in the medium. A yellow color throughout the medium denotes a negative ornithine result.

- 1. **Urea test**

**Preparation**

| **Ingredients** | **Composition** |
| --- | --- |
| Urea | 20 g |
| Disodium hydrogen phosphate | 9.5 g |
| Dipotassium hydrogen phosphate | 9.1 g |
| Yeast extract | 0.1 g |
| Phenol red | 0.01 g |
| PH | 6.8 at 25 ^0^C |

**Procedure**

1. A small amount of growth is harvested with a sterile (1 µL) loop or needle.
2. Lightly inoculated the surface of the agar slant.
3. Tubes are incubated under aerobic conditions at 36°C with caps loosened.
4. Tubes should be examined and results recorded at 24 hours, 48 hours, and 5-7 days.

**Interpretation of results**

**Positive-** intense pink color on the slant

**Negative-** no color change

- 1. **Simmons citrate agar**

**Preparation**

| **Ingredients** | **Gms/litre** |
| --- | --- |
| Magnesium sulphate | **0.2** |
| Ammonium dihydrogen phosphate | 1.0 |
| Dipotassium phosphate | 1.0 |
| Sodium citrate | 2.0 |
| Sodium chloride | 5.0 |
| Bromothymol blue | 0.08 |
| Agar | 15.0 |
| Final PH at 25 ^0^C | 6.8 |

**Procedure**

1. A small amount of growth was harvested with a sterile (1 µL) loop.
2. Lightly inoculated the surface of the agar slant.
3. Tubes were incubated under aerobic conditions at 36°C with caps loosened.
4. Tubes were examined and results recorded at 24 hours, 48 hours, and 3-5 days.

**Interpretation of results**

Positive - intense blue color (initially the color change may only occur on the agar slant)

Negative - agar remains green
