# Supplementary material for: Assessment of knowledge, practice, and status of food handlers toward Salmonella, Shigella, and intestinal parasites: A cross-sectional study in Tigrai prison centers, Ethiopia
Source: PLoS One. 2020 Nov 3;15(11):e0241145. doi: 10.1371/journal.pone.0241145 (PMC7608870; doi:10.1371/journal.pone.0241145)
Supplement: S1 File — (ZIP) [file pone.0241145.s001.zip › Questionnaire.docx]

Questionnaire on the assessment of knowledge and practice of food handlers on food borne pathogens at prison centers in Eastern Zone of Tigrai, 2019

**Socio-demographic characteristics of participants**

1. Gender: □ male □ female
2. Age: _____________
3. Level of education: □ Illiterate □ 1º school □ 2 º school □ College/ university
4. Marital status: □ single □ married □ divorced □ widowed
5. Work experience in food preparation (in months): ___________________
6. Job division: □ cleaning utensils □ food handling
7. Certified in food preparation: □ Yes □ No

**Food handling practice**

- 1. Always wear gown during food preparation: □ Yes □ No

1. Wear hair restraints during food preparation: □ Yes □ No
2. Wear finger ornaments during food preparation: □ Yes □ No
3. Cook/wash vegetables and fruits thoroughly before consumption: □ Yes □ No
4. Prepare food while you have diarrhea: □ Yes □ No
5. Always clean the work area before and after work: □ Yes □ No
6. Before you begin preparing food, how often do you wash your hands with soap?

All of the time Most of the time some of the time

Rarely Don't know

1. Wash hands with soap after using toilet: □ always □ sometimes □ never don’t know
2. Always wash hands after touching dirty materials: □ with soap and water □ with water only □ don’t wash
3. Food handlers touch their body during vending: □ Yes □ No
4. Always cut fingernails: Yes No

**Knowledge of food handlers**

1. Ever heard of food borne diseases: Yes No
2. Have you ever heard of any of the following as a problem in food?
   1. Salmonella Yes No
   2. Shigella Yes No
   3. Giardia Yes No
   4. Amoeba Yes No
3. Infected food handler transmit food borne diseases to the customers Yes No
4. Washing hands before work reduces the risk of food contamination. Yes No
5. Using gloves while handling food reduces the risk of food contamination Yes No
6. Proper cleaning and sanitization of utensils reduces the risk of food contamination. Yes No
7. Typhoid fever can be transmitted by food Yes No
8. Bloody diarrhea can be transmitted by food Yes No
9. Microbes are on the skin, in the nose and mouth of healthy food handlers. Yes No
10. Contaminated foods always have some change in color, odor or taste. Yes No
11. The health status of workers should be evaluated before employment Yes No
12. Rodents / vectors can spread food borne diseases. □ Yes □ No
